# Pathways of flower infection and pollen-mediated dispersion of *Pseudomonas syringae* pv. *actinidiae*, the causal agent of kiwifruit bacterial canker

**DOI:** 10.1038/s41438-018-0058-6

**Published:** 2018-11-01

**Authors:** Irene Donati, Antonio Cellini, Giampaolo Buriani, Sofia Mauri, Callum Kay, Gianni Tacconi, Francesco Spinelli

**Affiliations:** 1 Department of Agricultural and Food Sciences - DISTAL, Alma Mater Studiorum—University of Bologna, viale Fanin 44, 40127 Bologna, Italy; 2 ZESPRI GLOBAL Supply, 400 Maunganui Road, Mount Maunganui, New Zealand; 3 Consiglio per la Ricerca e la Sperimentazione in Agricoltura—Genomics Research Centre, via S. Protaso 302, 29017 Fiorenzuola d’Arda, Italy

## Abstract

Flowers can provide a protected and nutrient-rich environment to the epiphytic microflora, thus representing a sensible entry point for pathogens such as *Pseudomonas syringae* pv. *actinidiae* (Psa). This bacterium can colonize both male and female *Actinidia* flowers, causing flower browning and fall, and systemic invasion of the host plant, eventually leading to its death. However, the process of flower colonization and penetration into the host tissues has not yet been fully elucidated. In addition, the presence of Psa in the pollen from infected flowers, and the role of pollination in the spread of Psa requires confirmation.

The present study employed a Psa strain constitutively expressing the fluorescent GFPuv protein, to visualize in vivo flower colonization. Microscopy observations were performed by means of confocal laser scanning and wide-field fluorescent microscopy, and were coupled with the study of Psa population dynamics by quantitative PCR (q-PCR). The pathogen was shown to colonize stigmata, move along the stylar furrow, and penetrate the receptacles via the style or nectarhodes. Once the receptacle was invaded, the pathogen migrated along the flower pedicel and became systemic. Psa was also able to colonize the anthers epiphytically and endophytically. Infected male flowers produced contaminated pollen, which could transmit Psa to healthy plants. Finally, pollinators (*Apis mellifera* and *Bombus terrestris*) were studied in natural conditions, showing that, although they can be contaminated with Psa, the pathogen’s transmission via pollinators is contrasted by its short survival in the hive.

## Kiwi fruit: Insights to combat bacterial canker

Studying the transmission pathways of a bacterium that infects and kills kiwi fruit plants suggests that modified plant protection strategies could minimize the risk of disease. Researchers in Italy and New Zealand, led by Francesco Spinelli at the University of Bologna, investigated *Pseudomonas syringae pv. Actinidiae* (Psa), which causes kiwi fruit bacterial canker. This has been a major problem worldwide since a pandemic outbreak in 2008. The results show that flower tissues, especially the stigmata which receive transmitted pollen, are crucial sites allowing Psa to grow and penetrate into the plants. Evidence is presented of bacterial transmission via the pollen dispersed from plants that do not themselves show signs of infection. Suggested new control strategies include inspecting and treating pollen-donating plants, and using biological agents to compete with Psa growing inside kiwi fruit flowers.

## Introduction

Among the production constraints of kiwifruit, the bacterial canker, caused by *Pseudomonas syringae* pv*. actinidiae* (Psa), has been the major limiting factor in the main growing areas worldwide since its pandemic outbreak in 2008^1–3^. The disease affects various species of the genus *Actinidia*, including the two most important varieties for commercial purposes, *A. chinensis* var. *deliciosa* and *A. chinensis* var. *chinensis*^4–6^. In Italy, the first outbreak of bacterial canker was reported in 1994^7^, but serious economic damage started being observed with the spread of a highly virulent pathogen, named biovar 3, genetically separated from previously identified biovars^8–12^.

Many aspects of Psa biology and epidemiology still require in-depth investigation. Among them, the elucidation of the primary entry points and mechanisms of spread from plant to plant are of crucial importance. A few short communications have listed plant organs in which the pathogen was detected^13,14^. However, the detection of an endophytic pathogen within a specific plant organ, not accounting for its ability to move through plant tissues, does not imply a primary apoplastic invasion through the same organ. In addition, these studies were mainly performed in *A. chinensis* var. *chinensis*, allowing the isolation of Psa from a number of natural openings and wounds such as symptomatic lenticels, lesion margins, buds, fruit stalks, leaf petioles, and flowers^13,14^.

Typical flower symptoms consist of browning of petals and sepals in early stages of infection. Subsequently, Psa-infected blossoms may wither before opening, or fall off soon after fruit set. However, such symptoms are not specific to Psa, being similar to those of blossom blight caused by *Pseudomonas viridiflava* and *P. syringae* pv. *syringae*^15–18^.

Although female flowers can provide bacteria with a protected and nutrient-rich environment^14^, the process of flower colonization by Psa and its penetration into the host tissues has not been yet fully elucidated. The precise knowledge of the mechanisms and time of the invasion of the host tissue is essential to understand the disease cycle and to develop effective control strategies. Despite the fact that more than 100 bacterial species are known to be plant pathogens, pollen-mediated transmission of bacterial pathogens has only been postulated for *Xanthomonas arboricola* pv. *juglandis*^19,20^*, Erwinia amylovora*^21^ and *Pseudomonas syringae*^22^, even though they are not the only pathogens which may infect flowers, and compelling evidence for pollen-mediated bacterial spread in real conditions is still missing. In *Erwinia amylovora*, which is the most widely studied bacterial pathogen leading to systemic infection by flower colonization^23,24^, the production of contaminated pollen by infected flowers was achieved only in experimental conditions, by applying a highly concentrated (10^8^ cells mL^−1^) bacterial suspension directly on anthers^21^, while the production of contaminated pollen in natural conditions has never been described.

In addition, *E. amylovora* and other bacterial pathogens may rely on pollinating insects for secondary spread^25,26^. Several insect species, such as honeybees (*Apis mellifera*) and bumblebees (*Bombus* spp.), contribute to kiwifruit pollination^27^. However, although *Actinidia* spp. flowers are fragrant, their attractivity to pollinators is poor, and some of their characteristics (pendulous position, high pollen:ovule ratio, large multibranched stigmatic surfaces, scarce production of nectar) suggest that wind may be an important agent of pollen transfer^28,29^.

To obtain export-quality fruit, more than 2000 pollen grains should reach the stigma leading to fruits with 1000–1400 viable seeds^27^. Since pollination dictates the size and the quality of the kiwifruit berries, pollen collection and artificial pollination have become a widespread practice to enhance fruit production and size^30,31^.

Therefore, understanding the importance of pollen and pollination for the dissemination of Psa from plant to plant is a crucial step for the control of bacterial canker.

Several studies showed that *Actinidia* spp. pollen or commercial pollen preparations can be contaminated by Psa^13,32–36^. Preliminary results on the pollen-mediated vectoring of Psa were reported by Tontou et al*.*^37^. However, limited information is currently available on a number of crucial aspects regarding the actual presence of Psa in the pollen in field conditions, and the consequent epidemiological risk. In fact, although the viable Psa population on pollen grains was quantified in some experiments, crucial pieces of information (such as the minimal Psa population on pollen needed to effectively transfer Psa on flowers, the infection threshold of flowers, or the overall pollen-mediated infection efficacy) are still missing.

The present study elucidates the different phases of flower colonization by Psa till the systemic invasion of the host plant. Moreover, the effect of infection on fruit production and colonization by Psa was evaluated. Finally, this study provides the first evidence on the production of infected pollen by diseased male plants, and the role of pollen and pollination in the dissemination of Psa.

The colonization of the different flowers parts was studied in vivo and real-time by combining the use of a green fluorescent protein (GFP)-labeled Psa strain (CFBP7286-GFPuv) with fluorescent stereomicroscopy and confocal laser scanning microscopy. These methodologies allowed the univocal identification of the bacteria and the in vivo, non-invasive observation of plant tissues^13,24^. With the possible exception of fire blight^21,38^ (*E. amylovora*), this is the first, non-speculative report of pollen-mediated transmission of a phytopathogenic bacterium.

## Materials and methods

### Biological material

Experiments in controlled conditions were performed on kiwifruit cultivars Hayward (female, *A. chinensis* var. *deliciosa*), Tomuri (male, *A. chinensis* var. *deliciosa*), Hort16A (female, *A. chinensis* var. *chinensis*) and CK2 (male, *A. chinensis* var. *chinensis*), using 4-year-old potted plants maintained in plastic pots (40 cm diameter) with a mixture of peat and sand (1:1, v/v). Standard irrigation and fertilization was applied. As mineral fertilizer, Poly-Feed (16N-8P-32K) by Fertica S.A. was used (18 g per plant applied two times). During the experiments, the plants were kept in glasshouse conditions under natural light. Temperature and relative humidity were, respectively, 22–24 °C and 70–80%. After inoculation, the relative humidity was raised to 100% for the following 48 h.

The Psa strain CFBP7286-GFPuv was used for artificial inoculation. This genetically engineered strain, retaining the virulence of the corresponding wild type (CFBP7286, biovar 3), expresses a green fluorescent protein and kanamycin resistance^13^. The inocula were prepared by cultures grown in liquid Luria-Bertani medium under moderate shaking at 27 °C, then resuspending the bacteria in 10 mM MgSO_4_. The titer of bacterial suspensions was determined by plating 10-µL drops of serial 1:10 dilutions on Luria-Bertani medium containing 15 g L^−1^ of agar amended with cyclohexamide (100 mg L^−1^). In experiments using infected samples from commercial fields, Psa identity in serial dilutions was confirmed by q-PCR^39^.

### Experimental inoculation of *Actinidia* spp. flowers

Several inoculation methods of a Psa suspension at 10^6^ colony-forming units (cfu) mL^−1^ were used for each plant cultivar: (i) spraying the whole flower, (ii) dipping the stigmata or anthers in the bacterial suspension, or (iii) pipetting 20 µL of the same bacterial suspension on the calyx. Sixty flowers were inoculated with each of the three different inoculation methods. Water-treated flowers were used as the control. All the female flowers were artificially pollinated 6 h before inoculation.

The tissues (stigmata/anthers, ovarium, and other flower parts) of six spray-inoculated flowers were separated at 0, 3, 24, 48, 72, 96, and 120 h after inoculation, and the epiphytic Psa population was measured on them. The samples were vigorously washed for 5 min in 5 mL of sterile 10 mM MgSO_4_ solution.

To assess endophytic Psa populations, the same tissue samples were surface-sterilized by washing in ethanol (70%, 3 min) and NaOCl (0.1%, 3 min), followed by two rinses in sterilized water. Then they were homogenized in 10 mM MgSO_4_ and the bacterial population was assessed as previously described. The complete efficacy of the sterilization method was confirmed by comparing the epiphytic Psa population of unsterilized and sterilized organs. To assess Psa endophytic population in the flower-bearing cane (i.e., stalk), a wood section of 1 cm in length on either side of the flower pedicel was excised and analyzed. Each experiment was repeated three times.

### Psa presence on flowers and pollen in infected orchards

The sampling of flowers from male and female plants was performed in three commercial kiwifruit orchards in Faenza region, Italy (44°17'00.0“N 11°53'00.0“E), with a disease incidence of 50% (assessed as the proportion of vines showing symptoms).

The incidence of Psa and other kiwifruit pathogens (*P. syringae* pv. *syringae*, *P. viridiflava*) was determined on 600 samples of *A. chinensis* var. *chinensis* (cv. Hort16A) and *A. chinensis* var. *deliciosa* (cv. Hayward) female flowers showing browning symptoms. On flowers infected by Psa, the bacterial population of this pathogen was quantified.

The natural occurrence of Psa on asymptomatic male flowers and pollen was determined on closed (BBCH 53–55) and open flower samples (BBCH 60) (100 for each type and for the two *A. chinensis* varieties).

Anthers (from samples before dehiscence stage in closed flowers) or pollen (from fully open flowers) were aseptically separated from the remaining tissues of ten-flower samples. Before dissection of closed flowers, the external surface was sterilized as previously described. Pollen was washed with 20 mL of sterile water for 15 min on a rotary shaker at 120 rpm until full rehydration. Subsequently, the samples were precipitated by centrifugation (90 s at 10,000 × *g*), and the supernatant was used for bacterial enumeration.

Pollen germination was assessed as the percentage of granules emitting a tube after 5 h at 25 °C under shaded light, in a medium containing 10% sucrose and 15 g L^−1^ agar.

In 2016, to verify the possibility of contamination of pollen via an endophytic invasion, three 1-year -old *A. chinensis* var. *chinensis* potted male vines were spray-inoculated at bud break with CFBP7286-GFPuv (10^8^ CFU mL^−1^ in 10 mM MgSO_4_). Plants were kept in controlled conditions till flower development. At the beginning of flowering, ten opening flowers were collected from each plant and anthers were aseptically excised. Presence of Psa was assessed by serial dilution plating and q-PCR, as previously described.

### Flower colonization by pollen-vectored Psa

Pollen samples (0.5 g) of *A. chinensis* var. *chinensis* (cvs. CK2 and Belen) and *A. chinensis* var. *deliciosa* (cv. Tomuri) were artificially inoculated with 1.5 mL of a suspension of CFBP7286-GFPuv (10^7^ CFU mL^−1^ in 10 mM MgSO_4_) obtained from fresh cultures in liquid LB medium. The wet contaminated pollen was dried at room temperature for 72 h. For each pollen sample, the bacterial population was monitored over 5 days. Before using contaminated pollen for inoculation of flowers, Psa population was determined and adjusted to 4.1 × 10^6^ cfu g^−1^ by adding uncontaminated pollen. Pollen viability and germination were assessed as previously described.

Hand-pollination was performed on 4-year-old *A. chinensis* var. *chinensis* (cv. Hort16A) and *A. chinensis* var. *deliciosa* (cv. Hayward) potted plants, by dusting Psa-free pollen with a wet brush on each flower. In a second group of plants, infected pollen was used instead. Nine plants divided in three replicates were used for each treatment. Endophytic and epiphytic Psa populations were determined 5 and 10 days after inoculation in the different flower organs and in the bearing cane. The same experiments were repeated using naturally infected *A. chinensis* var. *chinensis* (cvs. CK2 and Belen) and *A. chinensis* var. *deliciosa* (cv. Tomuri) pollen. In this case, the Psa population was determined and adjusted to 1.2 × 10^4^ cfu g^−1^.

### Pollen-mediated vectoring of Psa in natural conditions

To mimic the pollen-mediated vectoring of Psa in natural conditions, flowering branches of male *A. chinensis* var. *chinensis* (cv. Belen) were detached from infected plants in a commercial orchard. Twelve branches were transferred in controlled semi-field conditions, maintained in water and placed for 5 days in proximity (1 m) to nine healthy *A. chinensis* var. *chinensis* (cv. Hort16A) potted plants divided in three replicates. To prevent uncontrolled diffusion of Psa in the environment by insects or water leach, plants were placed under a tunnel built with anti-aphid net (Antiafide 20/10 Mesh 50, Artes Politecnica s.r.l, Vicenza, Italy). The tunnel dimensions were 12 × 3.50 × 5.50 m. The floor was covered with an impermeable plastic film.

Psa load on naturally infected pollen was quantified as previously described, and it resulted to be 3 × 10^6^ cfu g^−1^.

A similar experiment was repeated on *A. chinensis* var. *deliciosa* (cv. Hayward) using infected Tomuri vines.

Incidence was calculated as the percentage of flowers presenting a systemic endophytic Psa population inside the stalk (i.e., flower-bearing canes). Epiphytic and endophytic Psa populations were determined 5 and 10 days after inoculation in the different flower organs and in the bearing cane.

### Systemic colonization of the host plant upon flower inoculation

To monitor the dynamics of systemic invasion upon flower infection, on six *A. chinensis* var. *chinensis* (cv. Hort16A) and six *A. chinensis* var. *deliciosa* (cv. Hayward) potted plants, a single flower cluster per plant was inoculated by using pollen from male plants of the same variety (cvs. Belen and Tomuri, respectively), contaminated by CFBP7286-GFPuv (4.1 × 10^4^ CFU g^−1^).

The evolution of flower symptoms, fruit set, development, and fall were monitored weekly. The endophytic migration of Psa was assessed 4 months after inoculation. Tissue samples were taken from cane tops, grafting points, rootstock wood and roots. Tissue samples were surface-sterilized by washing in ethanol (70%, 3 min) and NaOCl (0.1%, 3 min), followed by two rinses in sterilized water. Successively, they were homogenized in 10 mM MgSO_4_ and the bacterial population was assessed as previously described. The experiment was repeated twice.

### Psa vectoring by pollinators

The experiments were performed in 2 consecutive years in commercial orchards located in the area of Latina (41°28′03.35″N 12°54′13.32″E), with 15 to 20% disease incidence (assessed as the percentage of vines showing symptoms). Five commercial bumblebee hives (Natupol® Koppert B.V., The Netherlands) were placed in each orchard at 75% flowering. Five days after beehive positioning, bumblebee capture was performed for 3 consecutive days. Each sampling consisted of ten bumblebees captured after return to the beehive. Pollen harvested by bumblebees was also collected and analyzed. Psa was isolated and identified as previously described for other substrates.

To verify Psa survival on honeybees and in honeybee hives, a set of tubes was closed with microbiological filters. In each tube, a dead insect, 0.2 g wax, or 20 mg *A. chinensis* var. *deliciosa* (cv. Tomuri) pollen were placed, after inoculation with 10 µL of a Psa suspension with a bacterial titer of 10^6^, 10^8^ or 10^10^ cfu mL^−1^. The biological samples were maintained in a honeybee hive, together with a data logger recording temperature and relative humidity, and Psa population was monitored.

### Microscopy

Following inoculation with bacterial suspensions or with artificially contaminated pollen, Psa distribution over flower tissues, and migration through them was monitored by microscopical observation.

Sample preparation and initial observations were performed using a motorized Nikon SMZ25 (Nikon, Tokyo, Japan) with a zoom ratio of 25:1 (zoom range: 0.63 × - 31.45 × ) fitted with a BHS (GHS) filter set. The GHS filter set (excitation light 450–490 nm, emission 510 nm) was used to visualize the GFPuv-labeled bacteria.

Optical sections were obtained with a Nikon C1-S (Nikon, Tokyo, Japan) confocal laser scanning microscope (CLSM) and equipped with an Argon laser. A 40, 60 and 100 × Nikon PlanApo objectives and the BHS (GHS) filter set were used for image acquisition. Images were acquired and analyzed by the NIS-Elements C Microscope Imaging Software.

### Statistical analysis

Psa populations, at different time points and in different *A. chinensis* flowers parts, were analyzed by one-way analysis of variance (ANOVA) followed by means separation with Fisher’s least significant difference (LSD, *P* < 0.05). Incidence of male and female flowers contaminated by Psa in different *A. chinensis* variety was analyzed by *Z*-test (*P* < 0.05). For statistical analysis the software STATISTICA 7.0 (StatSoft Inc, Tulsa, USA) was used.

## Results

### Population of *Pseudomonas syringae* pv. *actinidiae* on *Actinidia* spp. flowers

**Fig. 1:**
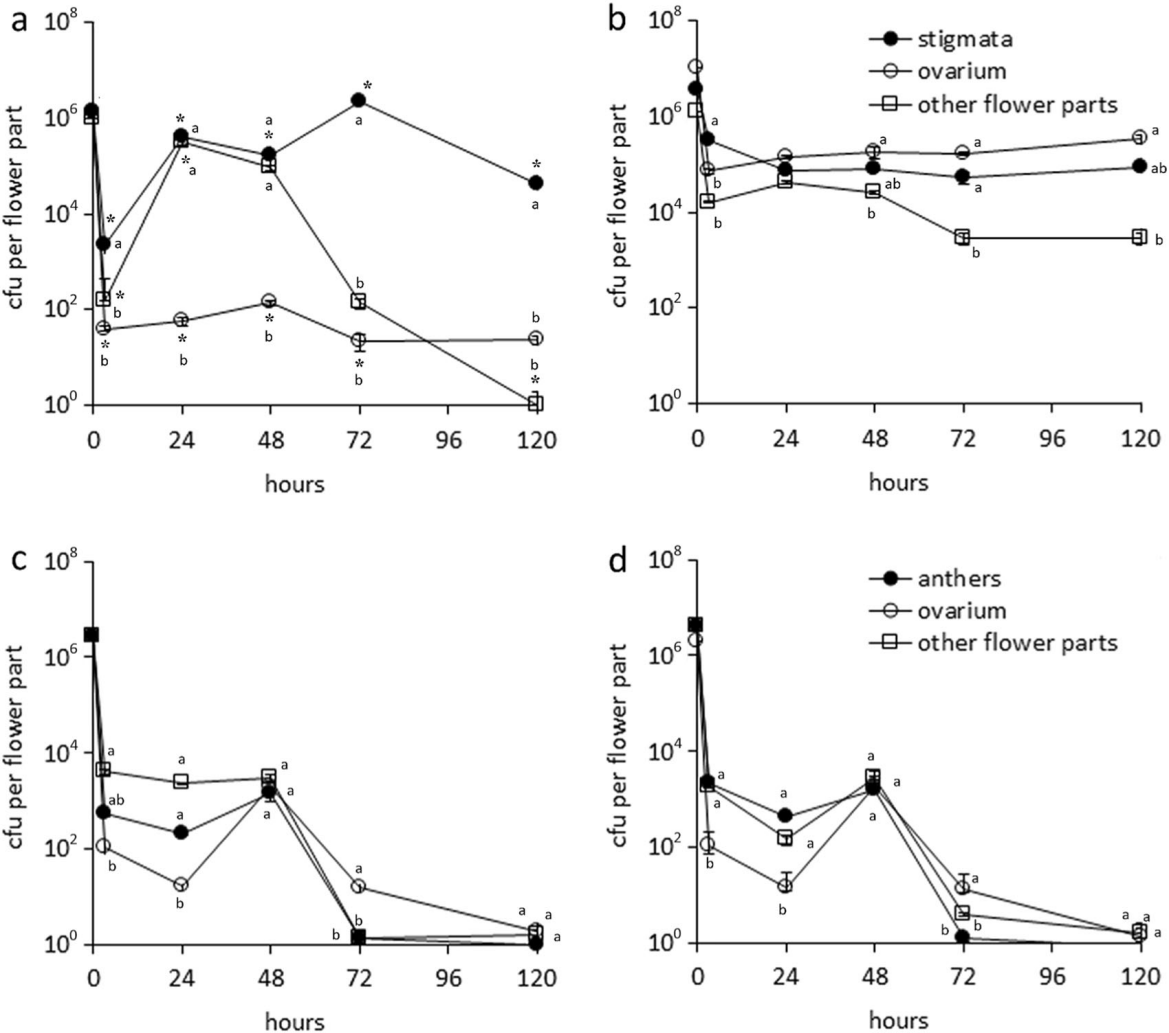
Evolution of *Pseudomonas syringae* pv. *actinidiae* (Psa) population over time in different *Actinidia chinensis* flower parts: **a** Female *A. chinensis* var. *deliciosa*, **b** female *A. chinensis* var. *chinensis*, **c** male *A. chinensis* var. *deliciosa,* and **d** male *A. chinensis* var. *chinensis*. Inoculation was performed by spraying flowers with a Psa (CFBP7286) suspension at a concentration of 10^6^ cells mL^−1^. Standard error is shown (*n* = 6 for each time point). Different letters indicate significant differences among flower parts within each variety at LSD test (*P* = 0.05). In female flowers, asterisk indicates significant differences of Psa population between the same flower part of *A. chinensis* var. *chinensis* and *A. chinensis* var. *deliciosa* (LSD test, *P* = 0.05). The same analysis performed on male flowers did not reveal any significant difference among varieties

Psa was able to establish a detectable population on all the flowers parts of both *A. chinensis* var. *chinensis* and *A. chinensis* var. *deliciosa* female flowers (Fig. 1a, b). The highest population was observed on stigmata and styles with an average value of 3.6 ± 2.1 × 10^5^ cfu for the whole duration of the experiment. Other flower parts, in *A. chinensis* var. *deliciosa*, hosted such a high population only transiently. In contrast, in *A. chinensis* var. *chinensis*, Psa population constantly ranged between 10^4^ and 10^6^ on all flower tissues (Fig. 1a, b). On male flowers, Psa attained a lower population, showing a substantial decrease after 48 h corresponding with tissue senescence (especially anthers; Fig. 1c, d). No differences were observed between *A. chinensis* var. *chinensis* and *A. chinensis* var. *deliciosa* male flowers.

The inoculation methods influenced the endophytic colonization of the different flower organs. Within floral tissues, the pathogen population was generally higher in *A. chinensis* var. *chinensis* tissues than in *A. chinensis* var. *deliciosa* (Supplementary Fig. S1). After inoculation by spray, the highest bacterial populations were found in the flower stalk. In female flowers, the dipping-inoculation of the stigma led to colonization of the flower pedicel and stalk only in *A. chinensis* var. *chinensis*, whereas on *A. chinensis* var. *deliciosa*, Psa was isolated from those flower parts only occasionally. After the infection of the ovarium, the pathogen was mainly found in the gynoecium and calyx (Supplementart Fig. S1).

On male flowers, the isolation of the pathogen from the flower tissues was achieved only after spray inoculation. Also on male flowers, the highest Psa populations were isolated from *A. chinensis* var. *chinensis*.

After spray inoculation, 75% of *A. chinensis* var. *chinensis* and 53% of *A. chinensis* var. *deliciosa* female flowers were infected *stricto sensu* (i.e., they harbored an endophytic Psa population). The infection of male flowers occurred, in both species, in approximately 60% of the inoculated samples.

**Fig. 2:**
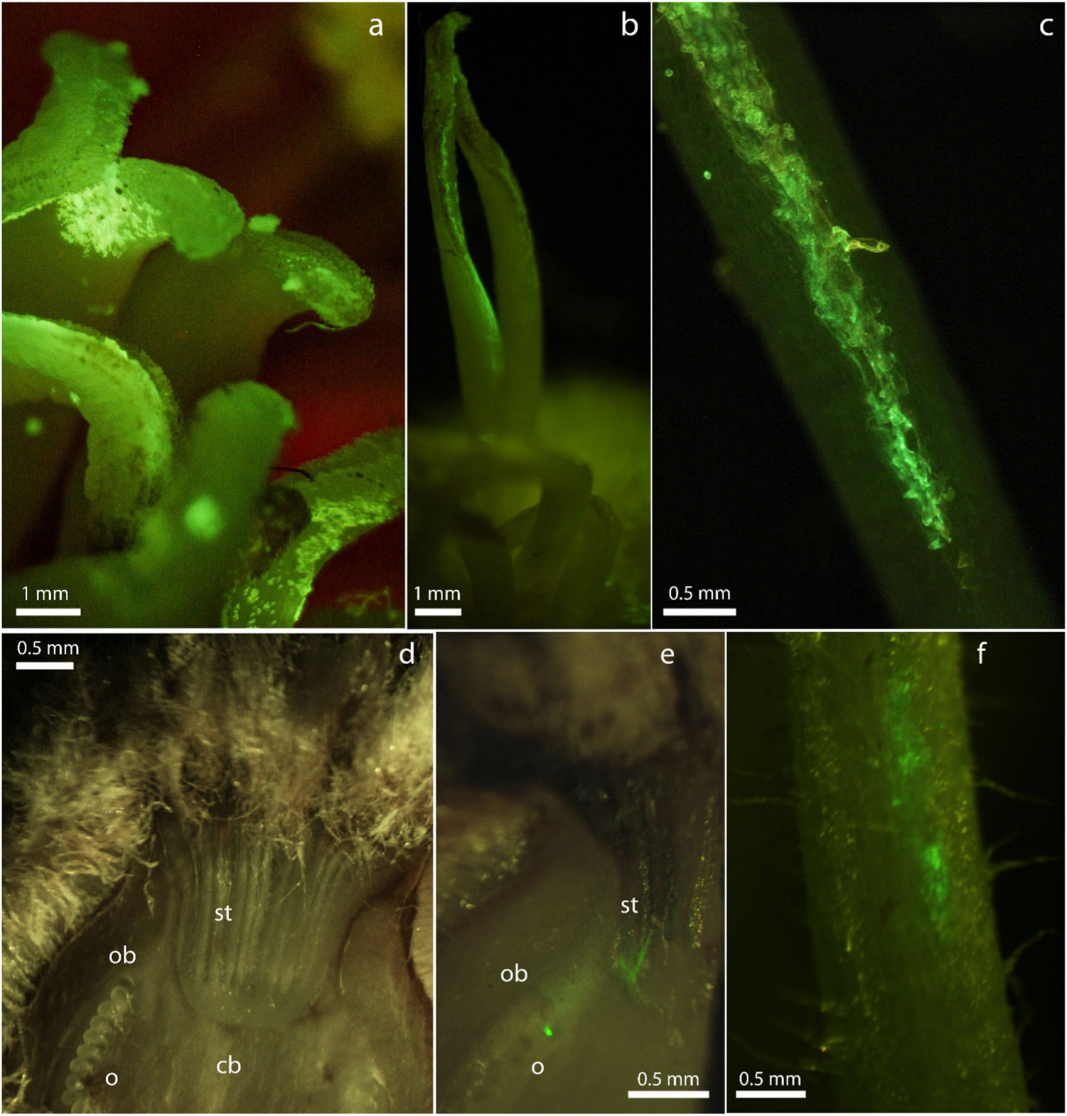
Fluorescent stereomicroscope photographs of *Actinidia chinensis* var. *deliciosa* flowers contaminated by *Pseudomonas syringae* pv. *actinidiae* (Psa) expressing a green fluorescent protein (CFBP7286-GFPuv). Inoculation was performed by pollinating stigmas of each flower with pollen contaminated by GFPuv-Psa. Bright green fluorescent emission is due to Psa CFBP7286-GFPuv colonization of tissues. Photographs were taken 24 h after inoculation (**a**, **b**, **c**) or 6–9 days after inoculation (**d**, **e**, **f**). **a** Stigma coated by contaminated pollen grains. **b, c** Psa CFBP7286-GFPuv migration along the whole stylar furrow to the ovarium (**d**–**e**). **d** Healthy ovarium. **e** Infected ovarium. The pathogen reached the ovarium via the styles (st). Central placenta vascular bundle (cb), ovary wall vascular bundle (ob) and ovules (o) are visible. Fluorescence is associated to the vascular bundles. **f** Flower pedicel, showing the high contamination by CFBP7286-GFPuv leading to a systemic invasion of from the ovarium into the flower-bearing cane. Bar measure is reported in each panel

**Fig. 3:**
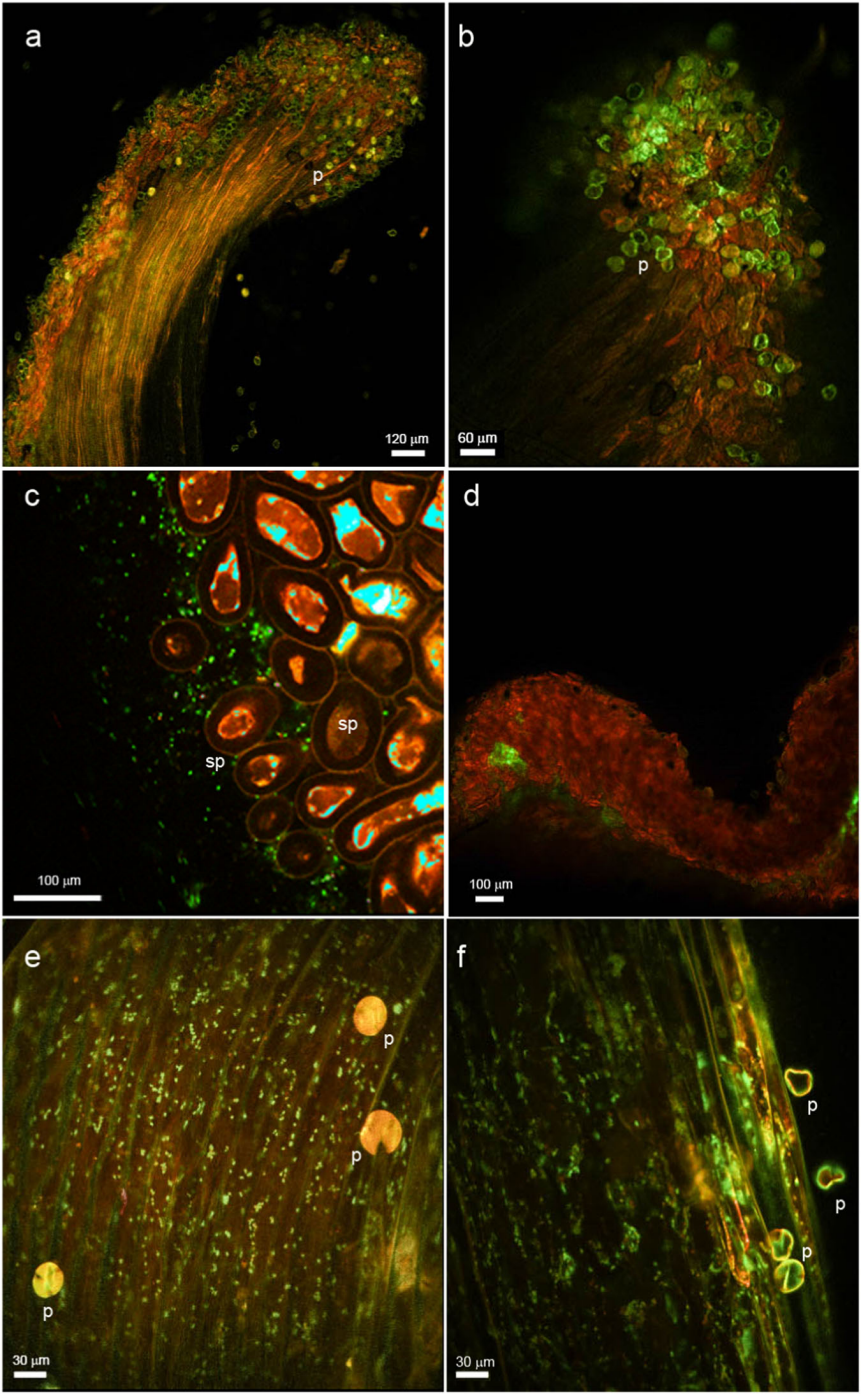
Confocal laser scanning (CLS) micrographs showing epiphytic colonization of kiwifruit (*Actinidia chinensis* var. *chinensis*) pistils by *Pseudomonas syringae* pv. *actinidiae* expressing a green fluorescent protein (CFBP7286-GFPuv) after pollination of the flowers with contaminated pollen. Photographs were taken between 24 and 36 h after inoculation. **a** *Actinidia chinensis* var. *chinensis* healthy stigma. Pollen grains (p) are visible and characterized by a green-yellow fluorescence emission. **b** Magnification of *Actinidia chinensis* var. *chinensis* stigma pollinated by Psa-contaminated pollen (p). The bright green fluorescent signal is due to CFBP7286-GFPuv. **c** *Actinidia chinensis* var. *deliciosa* stigma colonized by CFBP7286-GFPuv. Bright green rods represent single Psa cells. The brown-red, spherical structures are stigmatic papillae (sp). **d** *Actinidia chinensis* var. *deliciosa* stylar furrow heavily colonized by the pathogen. **e**, **f** *Actinidia chinensis* var. *chinensis* styles colonized by CFBP7286-GFPuv. Each green rod is a single pathogen cell. Pollen grains (p) show a roughly spherical shape and yellow fluorescence. Bar measure is reported in each panel

The microscopic observations show that the bacterium, after the colonization of the stigma, moves along the style and reaches the ovarium surface both in *A. chinensis* var. *deliciosa* (Fig. 2) and *A. chinensis* var. *chinensis* (Fig. 3). Psa can colonize the outer surfaces of the style by multiplying among the papillae and invading the stylar groove (Figs. 2b, c and 3d). However, it can also move along the style without a direct colonization of the stylar groove (Fig. 3e, f). Moreover, when bacteria establish an endophytic population, they can penetrate the conductive stylar tissue and move along the tracheids (Supplementary Fig. S2). On the ovarium surface, high pathogen populations were observed especially in *A. chinensis* var. *chinensis* (Fig. 1). In addition, penetration into the ovarium from the style has also been found (Fig. 2d, e).

### Growth of Psa on pollen and pollinated flowers

**Fig. 4:**
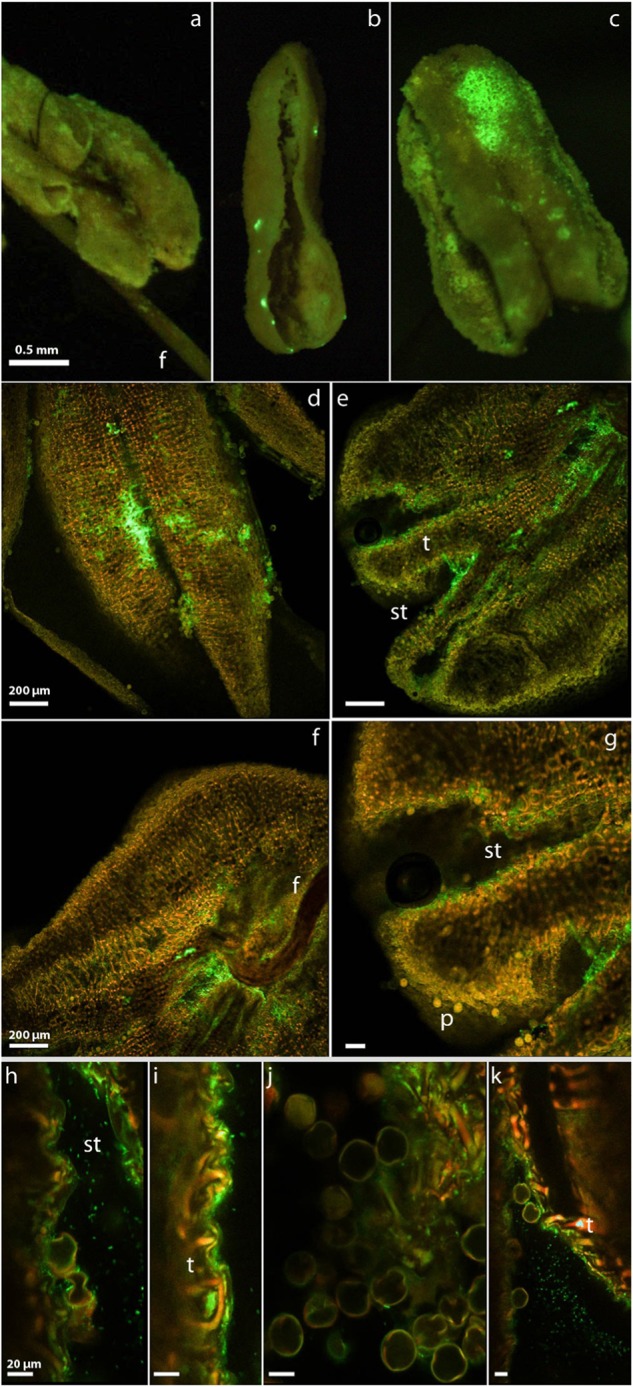
Colonization of anther and pollen grains of *A. chinensis* sp by *Pseudomonas syringae* pv. *actinidiae* expressing a green fluorescent protein (CFBP7286-GFPuv). Fluorescent steromicroscope (**a**–**c**) and confocal laser scanning (**f**–**k**) photographs of *Actinidia chinensis* var. *chinensis* anthers colonized by *Pseudomonas syringae* pv. *actinidiae* expressing a green fluorescent protein (CFBP7286-GFPuv). Inoculation was performed by spraying flowers with a suspension of CFBP7286-GFPuv cells. Photographs were taken 48 h after inoculation. **a** Healthy anther. The faint yellow-green signal is due to the natural fluorescence of pollen. After pollination of the flowers with contaminated pollen. **b** Dehiscent anthers showing epiphytic punctiform colonies of CFBP7286-GFPuv. **c** Anther with epiphytic aggregated colonies of CFBP7286-GFPuv. **d**–**g** Dehiscent anthers endophytically colonized by CFBP7286-GFPuv. The bacterial colonization of the endotecium is visible at dehiscence when pollen sacks open (**d**). The tapetum (t), which is the tissue responsible for pollen nutrition and maturation, is highly colonized by the pathogen (**e**). **f** The focus of infection is concentrated at the filament (**f**), suggesting that the pathogen reached the anthers by endophytic migration along vascular bundles. Stomia (st) are visible. **h**–**k** Magnification of a stomium showing pollen grains colonized by bacterial cells (**h**, **j**). **i** Collapsed cells of the tapetum (t) with extra- and endocellular colonization by CFBP7286-GFPuv. In all micrographs, each bright green, rod-shaped structure is a single CFBP7286-GFPuv cell. Bar measure is reported in each panel

The infection of anthers, even though not supporting higher pathogen populations than other tissues, resulted in contamination of pollen grains (Figs. 1c, d and 4). Anther colonization was often scattered, with small and dispersed areas of each anther colonized by the bacterium, possibly indicating an external, stochastic source of inoculum (Fig. 4b, c). In other cases, Psa colonization becomes visible only after anther dehiscence suggesting a possible internal infection of the anther via pathogen systemic migration along the filament (Fig. 4f). Whenever pollen contamination was observed, the infection of tapetum was also found (Fig. 4g–i). At dehiscence, infected anthers released in the stroma both contaminated pollen grains and free Psa cells (Fig. 4g, h, k). Only a small number of pollen grains were contaminated by Psa cells. In all these cases, Psa was found associated with the pollen grain surface (Fig. 4h–j). Infection of pollen grains (i.e., penetration of Psa into the microgametophytes) was never observed.

When spray-inoculated at bud break, before flower development, *A. chinensis* var. *chinensis* male vines produced infected anthers in (3 ± 1.2)% of flowers.

**Fig. 5:**
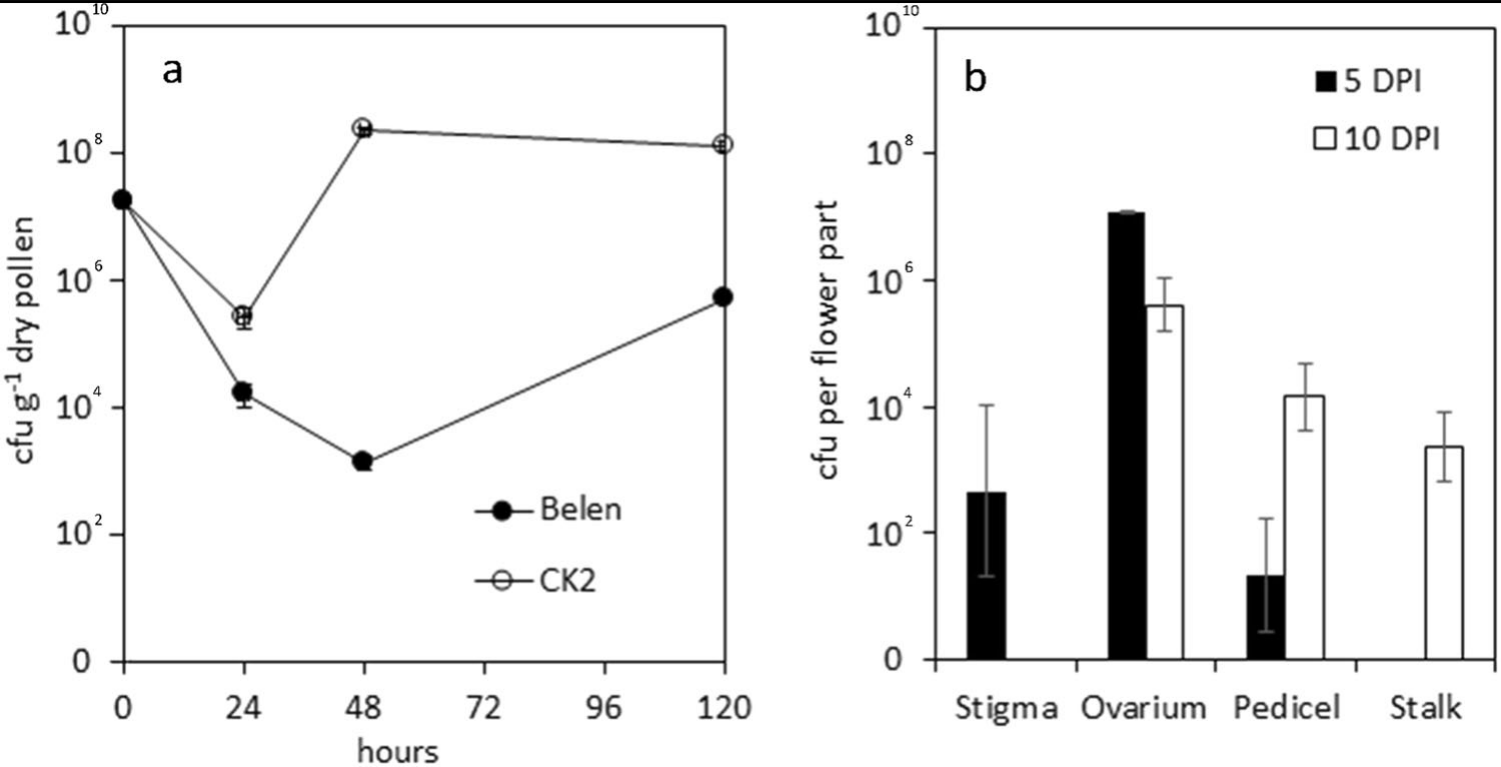
*Pseudomonas syringae* pv.* actinidiae* (Psa) persistence on pollen of* A*. *chinensis* var *chinensis* and pollen mediated colonization of different female flower organs. **a** Survival of *Pseudomonas syringae* pv. *actinidiae* (Psa) on pollen from *Actinidia chinensis* var. *chinensis* (cvs. Belen and CK2) (*n*  =  6 for each time point, per cultivar). **b** Bacterial population on female *A. chinensis* var. *chinensis* flower parts, assessed 5 (*n* = 9) and 10 (*n* = 6) days post inoculation (DPI) with Psa-contaminated pollen. Standard error is shown

Psa was able to survive on pollen grains (Fig. 5a) for at least 5 days, as confirmed by microscopical observation. After the pollination of *A. chinensis* var. *chinensis* flowers with infected pollen, Psa initially colonized the stigma and, thereafter, all the floral tissues, reaching the stalk in less than 10 days (Fig. 5b).

Following the exposure of healthy *A. chinensis* var. *chinensis* plants to infected male vines, the natural inoculation via contaminated pollen led, in 10 days, to 100% of contaminated flowers with (48 ± 12)% of them resulting in systemic invasion of the plant with an average endophytic Psa population of 10^4^ cfu g^−1^ FW.

In *A. chinensis* var. *deliciosa*, natural inoculation by contaminated pollen resulted in (44 ± 18)% of flowers harboring an epiphytic Psa population, whereas (25 ± 13)% of flower infections led to systemic invasion.

### Systemic colonization of plant tissues after pollen-mediated inoculation

After the growth on stigma and stylar furrow, which offers a nourishing and protected environment, Psa established an endophytic population inside the style (Supplementary Fig. S2 and Fig. 5) and the ovarium (Figs. 2 and 5). In particular, Psa was localized at the conjunction between the conductive stylar tissue and the central placenta, thus suggesting the penetration of the ovarium via migration inside the style. Penetration via nectarthodes was not observed in these experiments. Once inside the ovarium, Psa can colonize the vascular bundles, which may lead to the migration to flower pedicels. The migration of Psa via flower pedicel was observed by microscopical analysis (Fig. 2f) and resulted in systemic infection of the plant (Fig. 5b). Leaf spots were observed 2 months after flower inoculation.

In rare cases, highly infected female flowers produced droplets of bacterial exudate emerging with the nectar. Under high humidity conditions, the bacterial exudates dropped along the pedicel till reaching the abscission zone which, at flower drop, may represent a possible entry point.

Four months after inoculation of a single flower, the bacterium could be found in all the tissues (canes, grafting point, rootstock, roots), independent of the source of pollen infection (natural or experimental) and its bacterial load (Table 1), showing both acropetal and basipetal migration.

**Table 1:** Average *Pseudomonas syringae* pv. *actinidiae* (Psa) populations in several tissues of *Actinidia chinensis* var. *chinensis*, expressed in log (cfu g^-1^ plant tissue), 4 months after inoculation of a single flower with contaminated pollen

	Psa population, log (cfu g^−1^ plant tissue)			
	Apex (100%)	Graft point (75%)	Rootstock (75%)	Roots (75%)
Naturally contaminated pollen (4.1 × 10^4^ cfu g^−1^)	2.9 ± 1.4	5.4 ± 1.8	7.6 ± 1.4	6.9 ± 0.6
Experimentally contaminated pollen (10^6^ cfu g^−1^)	5.7 ± 2.0	5.3 ± 2.7	5.5 ± 2.7	5.0 ± 2.6

The pathogen migrated systemically in all infected plants (Disease Incidence = 100%). The relative incidence of Psa in the different plant organs is reported in brackets. Standard error is shown (*n* = 6)

In the same plants, all fruit dropped within 75 days after flowering, when the fruit diameter was below 40 mm. In contrast, control (healthy) plants only dropped 21% of their fruit load in 84 days (end of monitoring period).

### Flower blight incidence and contamination of pollinators in infected orchards

On *A. chinensis* var. *chinensis* female flower samples showing browning symptoms, Psa incidence was 82%, with an average population of 3.3 ± 1.6 × 10^8^ cfu per flower. In contrast, 17% of these flowers were infected with either *P. syringae* pv. *syringae* or *P. viridiflava*. Psa was also found on 40% of asymptomatic flowers, but with an average population 1000 times lower than in symptomatic flowers (Table 2). The Psa incidence and epiphytic population were substantially similar between *A. chinensis* var. *chinensis* and var. *deliciosa* female flowers (Table 2).

**Table 2:** Incidence of male and female flower contamination and *Pseudomonas syringae* pv. *actinidiae* (Psa) average population in *Actinidia chinensis* var. *chinensis* and *A. chinensis* var. *deliciosa* (100 flowers per each variety) collected from symptomatic plants

	Category	Flowers		Pollen	
		Incidence (%)	Population (cfu per flower)	Incidence (%)	Population (cfu g^−1^)
Male					
* A. chinensis* var. *chinensis*	Closed	71 b*	(2.9 ± 0.8) × 10^4^	5 a	(1.9 ± 0.9) × 10^2^
	Open	56 a*	(2.8 ± 0.7) × 10^8^	10 a*	(2.6 ± 1.1) × 10^2^
* A. chinensis* var. *deliciosa*	Closed	56 a	(2.9 ± 1.5) × 10^4^	4 a	(9.9 ± 2.5) × 10^1^
	Open	100 b	(7.8 ± 4.4) × 10^4^	80 b	(2.5 ± 0.7) x 10^2^
Female					
* A. chinensis* var. *chinensis*	Asymptomatic	40 a	(5.9 ± 1.2) × 10^5^	‒	‒
	Symptomatic	82 b	(3.3 ± 1.6) × 10^8^		
* A. chinensis* var. *deliciosa*	Asymptomatic	36 a	(1.5 ± 0.9) × 10^5^	‒	‒
	Symptomatic	73 b	(2.7 ± 1.7) × 10^8^		

Flowers were grouped according to either their symptomatology (asymptomatic or browning) or phenological stage (closed or open). Standard error is shown. Incidence values followed by different letters are significantly different according to *Z*-test (*P* < 0.05). Lowercase letters indicate difference between closed and open or asymptomatic and symptomatic flowers, *indicates significant differences between varieties for same kind of flowers

On male flowers, anthers (from closed flowers) and pollen (from open ones) were separately analyzed and Psa was detected in 56% and 100% of the open flowers in *A. chinensis* var. *chinensis* var. *deliciosa*, respectively (Table 2). In closed flowers, the presence of Psa is limited to the outer side of the flower, while the anthers are generally not reached by the bacterium (Table 2). However, Psa was found in the anthers or pollen with a significant bacterial population (average 2.2 ± 1.3 × 10^2^ cfu g^−1^), independently of flower phenological stage and presence of symptoms. This pollen was characterized by a very low viability, in no case higher than 5%.

During the 3 years of monitoring (2014–2016) of infected *A. chinensis* var. *deliciosa* orchards, about 20% of the worker bumblebees were contaminated with Psa (Table 3). On these individuals, the average Psa population in the different years was 2.8 ± 2.6 × 10^3^ cells per insect. Significant Psa populations were also detected on the pollen harvested by bumblebees and stored in the hive (Table 3). About 40% of the pollen samples were contaminated by Psa (Table 3). On experimentally inoculated honeybee bodies, wax, and pollen maintained in a honeybee hive, viable Psa units were not detected 48 h after inoculation.

**Table 3:** *Pseudomonas syringae* pv. *actinidiae* (Psa) incidence and average population on bumblebees (*Bombus terrestris*) and harvested pollen samples collected during kiwifruit flowering over 3 years (2014–2016)

Year	Bumblebees		Pollen	
	Incidence (%)	Population (cfu per insect^−1^)	Incidence (%)	Population (cfu g^−1^)
2014	20	(4.3 ± 0.7) × 10^3^	40	(3.4 ± 0.1) × 10^2^
2015	50	(3.3 ± 2.3) × 10^3^	50	(5.9 ± 0.9) × 10^4^
2016	20	(6.7 ± 5.0) × 10^2^	30	(1.2 ± 0.0) × 10^4^

The experiment was performed in a commercial *Actinidia chinensis* var. *chinensis* orchard. Bumblebees capture was performed during 3 consecutive days, and, in each day, ten individuals were captured at return to the beehive (*n* = 30). Standard error is shown

## Discussion

### Colonization of flowers by Psa

Psa was able to densely colonize flowers. The styles hosted the highest populations ( ≥ 10^5^ cfu), possibly because of nutrients and moisture secreted by the stigma. Male flowers are less conducive for Psa growth.

*A. chinensis* var. *chinensis* female flowers supported more uniform bacterial populations in the different flower parts, compared to *A. chinensis* var. *deliciosa* (Fig. 1). Since Psa attains similar populations on the styles of both *A. chinensis* varieties, the chemical composition of flower secretions is probably equally favorable to Psa growth. Morphological differences may explain the more widespread colonization of Psa on *A. chinensis* var. *chinensis*. In this variety, the denser trichome coverage on leaves, shoots, and flowers may promote moisture preservation, which is a key factor for flower-colonizing bacterial communities^40^. Furthermore, trichomes may also represent an entry point for the pathogen^13^.

Contamination of male flowers also resulted in the production of contaminated pollen. The contamination is substantially lower in closed than in open flowers, suggesting that pollen is generally contaminated by external inoculum sources (e.g., rain, wind, insects). However, contaminated pollen was also found in 5–10% of the closed flowers, where an external source of contamination can be excluded. Moreover, the experiment performed by inoculating, before flowering, male vines with CFBP7286-GFPuv confirmed this finding. Therefore, a systemic acropetal migration of Psa to anthers from other flower tissues could be responsible for this result. Nonetheless, an epiphytic contamination at bud break of developing sepals and petals and the migration of the pathogen to the inner tissues of flowers till reaching the anthers cannot be completely excluded. Finally, Psa populations on pollen were independent to the Psa population on flowers.

The apparently different incidence of Psa in open *A. chinensis* var. *chinensis* and *A. chinensis* var. *deliciosa* male flowers and pollen is probably due to a different disease pressure inside the orchards and to the different climatic conditions at flowering. In fact, *A. chinensis* var. *deliciosa* flowering takes place approximately 20 days after *A. chinensis* var. *chinensis*, when the climatic conditions (higher temperatures, lower relative humidity, lower occurrence of rainfall, and leaf wetting) may be less conducive for epiphytic Psa growth.

### Invasion of host tissues

Unlike fungal pathogens, bacteria are generally unable to actively penetrate plant tissues, and rely on wounds and natural openings, such as stomata, lenticels, and nectarthodes^41^ to access the host’s apoplast. Flowers have been previously suggested to be among the major entry points for Psa, although no experimental evidence was available^42–44^. In this view, flower infection would lead to systemic invasion of the whole plant.

In this work, high bacterial populations, showing that Psa was multiplying, were found on stigmata, suggesting that the stigma is a strong candidate for initial flower infection. Supporting this view, the direct dip-inoculation of stigmata caused the highest pathogen population within the flower pedicel. Subsequently, the bacterium moves along the stylar furrow and stylar connections toward the ovarium, where further multiplication and penetration to the apoplast may occur. The ovarium surface is, in fact, rich in trichomes and represents a suitable environment for the pathogen. In contrast, direct penetration from the calyx into the ovarium is unlikely, since there are no natural connections between these organs.

Bacterial plant pathogens may have an obligate infection path through flower tissues. *E. amylovora*, for instance, migrates along the style and the stylar furrow^24^ before accessing the ovary tissue. In contrast, Psa can also penetrate into the apoplast from the stylar surface (Supplementary Fig. S2). In this case, the bacterium was found in the conductive tissues, but not in the tracheids. This observation may suggest that the pathogen is unable to move through the xylem sieves of small vessels.

Further migration to the flower pedicel and flower stalk has also been observed. The invasion of flower stalks eventually leads to systemic infection.

Male flowers collected in the field may also be infected by the pathogen, with similar susceptibility and symptomatology to female flowers. However, the infection path through their tissues is not as clear as in female flowers. Since the presence of Psa in the flower stalk (Fig. 1) may be due to accidental contamination of the stalk itself during the experimental inoculation, the systemic penetration of the bacterium via male flowers still needs further verification.

### Ecological significance of pollen-mediated Psa spread and role of pollinators

Closed, asymptomatic flowers collected from symptomatic plants showed to be infected with Psa and to produce contaminated pollen, thus demonstrating that Psa may endophytically migrate to developing flowers in infected plants, leading to the contamination of anthers and the production of contaminated pollen. Paradoxically, Psa was more frequently found (although with lower populations) in asymptomatic flowers, and on pollen derived from asymptomatic flowers, than on symptomatic ones. It may be suggested that symptomatic flowers drop from the vine as its metabolism is no longer effective. This hypothesis is further supported by the observation that, in controlled conditions, all flowers infected via contaminated pollen dropped within 75 days after pollination.

Evidence of pollen contamination with Psa was previously provided^32,36^. However, the attempts to establish the ecological significance of pollen-vectored Psa in the development of bacterial canker^33,37^ only led to circumstantial evidence, either because of the artificial conditions of laboratory experiments, or because of the inability to track the source of infection and the bacterium during the various stages of pathogenesis in field conditions. Crucial information was missing about ecological aspects (such survival on flower parts and pathogen infection pathways), presumably affecting the chance of pollen-mediated Psa spread in field conditions.

Our research aimed to fill the information gap between flower inoculation and whole plant colonization, by visualizing the infection path exploited by Psa to penetrate the host plant’s apoplast. Our observations strongly suggest pollen-mediated flower contamination as one of the main infection pathways in field conditions.

Psa was found to survive on honeybees and in hives^45^. However, the importance of honeybee-mediated infection in field conditions is not clear. In this work, limited survival of Psa was observed in hive conditions; notably, the initial population in each sample had little influence on the survival time, suggesting that hive temperature (35 °C) and/or antimicrobial compounds, naturally present on the biological samples taken from the hive, may prevent Psa growth^34^. In addition to honeybees, significant Psa populations were also found on bumblebees foraging in infected fields. Overall, although direct evidence for insect-mediated Psa vectoring is still missing, the bacterial populations in hives and on insects are compatible with those employed for successful plant inoculation by hand-pollination (approx. 10^4^ cfu g^−1^ pollen), but only for a short time after contamination. Thus, pollinators may be involved in short-range, secondary Psa spread.

## Conclusions

This work demonstrates that flower tissues, and particularly stigmata, are crucial sites for Psa growth and penetration into the host tissues. Moreover, our work reports the first evidence of production of contaminated pollen by asymptomatic closed male flowers from infected plants and the role of pollen-mediated dispersal of a bacterial plant pathogen in natural conditions. Thus, plant protection strategies should consider the cultural practices involving flowers (for instance, inspection of pollen-donor plants, sanitation of pollinators, or the use of biological control agents competing with Psa in the floral niche) to minimize the risk of disease.

## Electronic supplementary material

Supplemmentary information
