# Supplementary material for: Pathways of flower infection and pollen-mediated dispersion of Pseudomonas syringae pv. actinidiae, the causal agent of kiwifruit bacterial canker
Source: Hortic Res. 2018 Nov 1;5:56. doi: 10.1038/s41438-018-0058-6 (PMC6210195; doi:10.1038/s41438-018-0058-6)
Supplement: Supplementary file 1 — Supplemmentary information [file 41438_2018_58_MOESM1_ESM.docx]

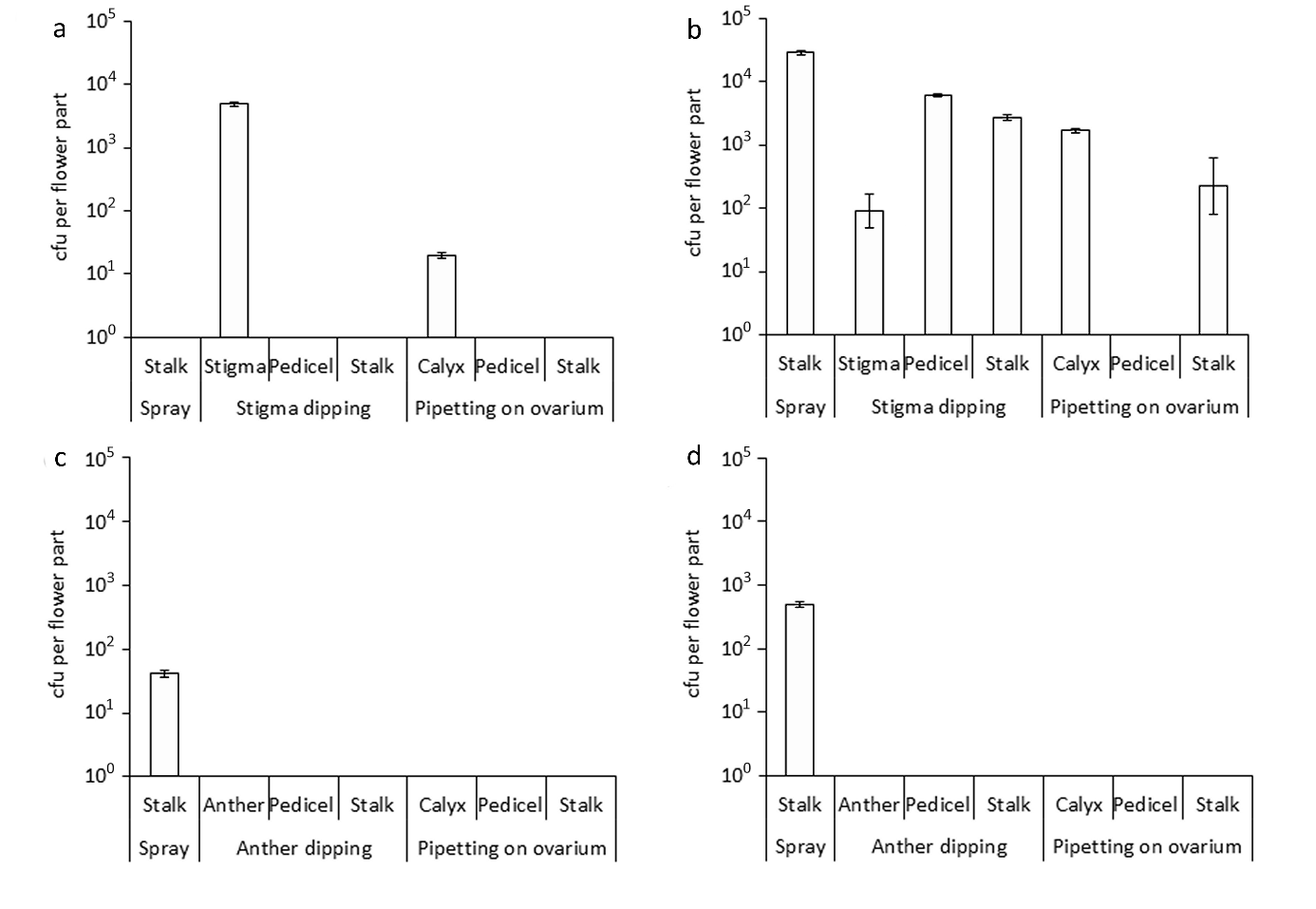


**Figure S1**

Psa population in different *Actinidia* *chinensis* flower parts, according to different inoculation methods (bacterial suspension spray, stigma/anther dipping, or pipetting in the calyx). (a) female *A. chinensis* var. *deliciosa*; (b) female *A. chinensis* var. *chinensis*; (c) male *A. chinensis* var. *deliciosa*; (d) male *A. chinensis* var. *chinensis*. Standard error is shown.


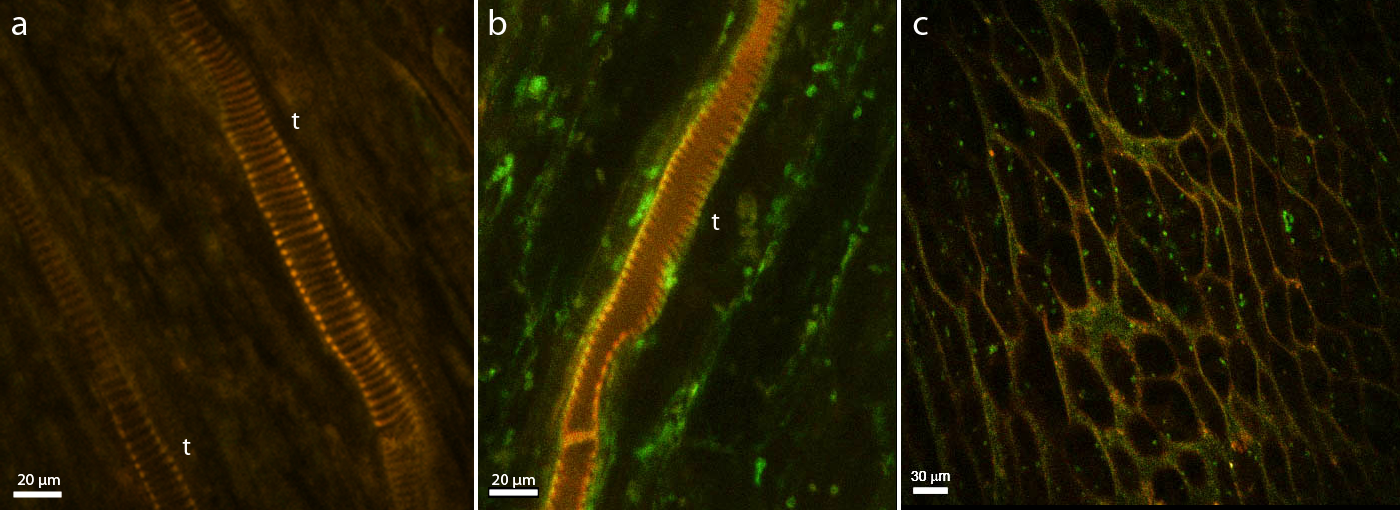


**Figure S2.**

Confocal laser scanning (CLS) micrographs showing endophytic colonization of *A. chinensis* var. *chinensis* pistils by GFPuv-*Pseudomonas syringae* pv. *actinidiae* (CFBP7286-GFPuv) after pollination of the flowers with contaminated pollen. (a) Healthy pistil with visible tracheids (t) characterized by the helical structure. (b) Infected pistil. Each green rod-shaped structure is a GFPuv-Psa cell. Bacteria are found associated to tracheids. (c) Colonization of the transmitting tissue. Each green, rod-shaped structure is a GFPuv-Psa cell. Bar measure is reported in each panel.
